# Gene editing of the extra domain A positive fibronectin in various tumors, amplified the effects of CRISPR/Cas system on the inhibition of tumor progression

**DOI:** 10.18632/oncotarget.21136

**Published:** 2017-09-21

**Authors:** Wan-Qi Lv, Hai-Cheng Wang, Jing Peng, Yi-Xiang Wang, Jiu-Hui Jiang, Cui-Ying Li

**Affiliations:** ^1^ Central Laboratory, Peking University School and Hospital of Stomatology, Beijing 100081, China; ^2^ Department of Pathology, School & Hospital of Stomatology, Tongji University, Shanghai Engineering Research Center of Tooth Restoration and Regeneration, Shanghai 200072, China; ^3^ Department of Beijing Citident Stomatology Hospital, Beijing 100032, China; ^4^ Department of Orthodontics, Peking University School and Hospital of Stomatology, Beijing 100081, China

**Keywords:** CRISPR/Cas, extracellular matrix, extra domain A fibronectin, gene editing, tumor progression

## Abstract

**Background:**

The low efficiency of clustered, regularly interspaced, palindromic repeats-associated Cas (CRISPR/Cas) system editing genes *in vivo* limits the application. A components of the extracellular matrix (ECM), the extra domain A positive fibronectin (EDA+FN), may be a target for CRISPR/Cas system for the pro-oncogenic effects. The exclusion of EDA exon would alter the microenvironment and inhibit tumor progression, even the frequency of gene editing is still limited.

**Results:**

The pro-oncogenic effects were confirmed by the exclusion of EDA exon from the fibronectin gene, as illustrated by the down-regulated proliferation, migration and invasion of CNE-2Z or SW480 cells (P<0.05). Furthermore, although the efficacy of EDA exon knockout through CRISPR/Cas system was shown to be low *in vivo*, the EDA+FN protein levels decrease obviously, inhibiting the tumor growth rate significantly (P<0.05), which was accompanied by a decrease in Ki-67 expression and microvessel numbers, and increased E-cadherin or decreased Vimentin expression (P<0.05).

**Methods and materials:**

Human nasopharyngeal carcinoma cell line CNE-2Z, and the colorectal carcinoma cell line SW480 were transfected with CRISPR/Cas9 plasmids targeting EDA exon. The effects of the exclusion of EDA on the cell proliferation, motility and epithelial-mesenchymal transition (EMT) were investigated, and the western blot and real-time PCR were performed to analyze the underlying mechanisms. Furthermore, CRISPR/Cas9 plasmids were injected into xenograft tumors to knockout EDA exon *in vivo*, and tumor growth, cell proliferation, EMT rate, or vascularization were investigated using western blot, PCR and immunohistochemistry.

**Conclusion:**

CRISPR/Cas system targeting ECM components was shown to be an effective method for the inhibition of tumor progression, as these paracrine or autocrine molecules are necessary for various tumor cells. This may represent a novel strategy for overcoming the drug evasion or resistance, in addition, circumventing the low efficiency of CRISPR/Cas system *in vivo*.

## INTRODUCTION

The conventional treatment strategies attempting to eradicate tumor cells as radical as possible, are frequently inefficient, because of the evasion from drugs, where the drug-sensitive tumor cells are eliminated, but the pre-existing insensitive sub-clones are selected and are able to survive [1, 2]; and drug resistance development represents an evolution of cancer cells, through mutations or reprogramming metabolic patterns due to the unstable genomes [3, 4]. As a consequence of drug evasion or resistance development, cancer therapy fails and leads to relapse [5]. In addition, the application of personalized medicine is limited by the lack of defined driver mutations, except in those patients carrying the genetic sequences that match the targeted drugs [5, 6]. For example, only 30% of mammary adenoid cystic carcinoma can be attributed to MYB/NFIB fusion, while the driver mutations in other cases remain unclear [7]. Therefore, the drug evasion, resistance development and the lack of defined therapeutic targets limit the development of efficient cancer therapies.

To some extent, tumors can be considered ecological systems in which Darwinian evolution applies [2, 5], and therefore, the modification of the tumor microenvironment may be an alternative approach to the inhibition of tumor progression [8], in order to prolong the survival periods. In tumor microenvironment, paracrine or autocrine molecules participate in maintaining favorable conditions for the propagation of tumor cells, such as the extracellular matrix (ECM) component extra domain A positive fibronectin (EDA+FN), as well as the vascular endothelial growth factor A isoforms (*VEGF*-Axxx), the pro-oncogenic isoforms generated by the alternative splicing of FN or VEGF genes [9, 10], respectively. These alternative spliced isoforms are always absent in normal adult tissues, but exclusively expressed in tumor, wound healing and inflammation. On this basis, most types of tumor cells could attenuate the physical barriers formed by the ECM [11, 12], or vascularize the tumor tissue in advance [10]. By targeting those paracrine or autocrine molecules spreading over the tumor microenvironment, it may not be necessary to eradicate all tumor cells, but modify a part of them, primarily the sub-populations supporting the propagation of the whole community [13]. The tumor recurrence can perhaps be avoided as well, because of the lack of survival pressure which facilitates the drug evasion or resistance development [14].

Using the type II bacterial clustered, regularly interspaced, palindromic repeats (CRISPR)-associated (Cas) system, the sub-population supporting the progression of tumors via their paracrine or autocrine molecules could be altered at the level of genome [15]. Although the limited efficiency of gene editing *in vivo* made it difficult to acquired therapeutic effects by targeting some oncogenes [16, 17], targeting paracrine or autocrine molecules using CRISPR/Cas system may be a strategy for amplifying the inhibition of tumor propagation, considering the diffusion of these molecules, and hence it is unnecessary to access each tumor cells. In this study, we confirmed the pro-oncogenic effects of EDA+FN, and the EDA exon-eliminating CRISPR/Cas9 plasmids were used both *in vitro* and *in vivo* to inhibit various tumor progression, based on our previous study [15].

## RESULTS

### *In vitro* EDA exon knockout with CRISPR/Cas system

**Figure 1:**
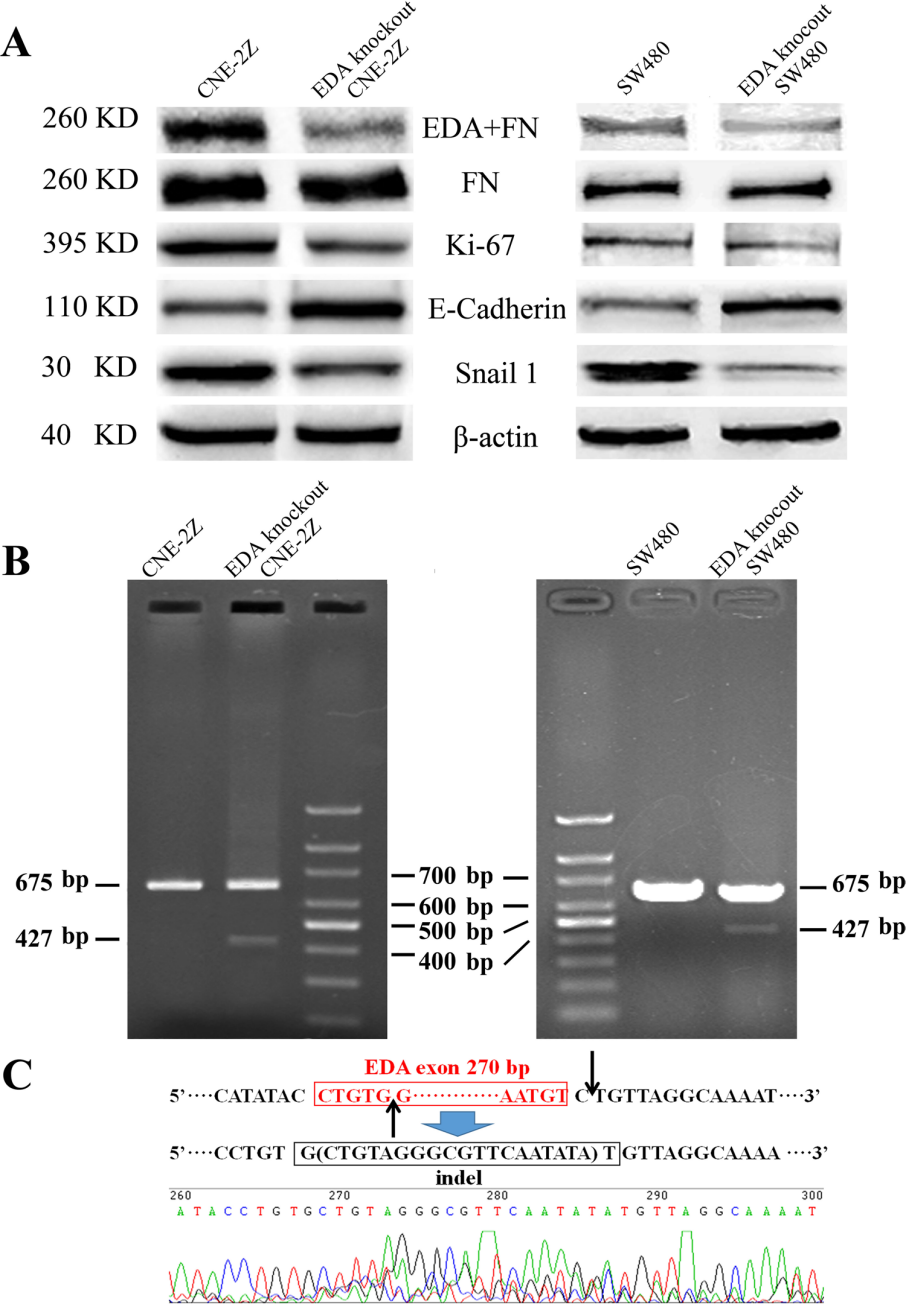
**(A)** Western blot analysis of EDA+FN and the total FN levels, as well as Ki-67, E-cadherin, and Snail 1 levels in the untreated and EDA knockout cells, the CNE-2Z and SW480 were presented respectively. **(B)** The PCR products of the genome isolated from EDA knockout and untreated cells, including CNE-2Z and SW480. **(C)** DNA sequencing of the PCR product at the length of 427 base pairs (bp), demonstrating EDA exon is removed in part of EDA knockout cells.

Compared to the untreated CNE-2Z or SW480 cells respectively, the levels of EDA+FN protein were shown to be significantly decreased in each cell line, after tansfecting with CRISPR/Cas plasmids targeting EDA exon; and in accordance with a previous study [15], the amount of total FN remained almost unchanged (Figure 1A). FN gene was amplified in EDA knockout CNE-2Z or SW480 cells respectively, a relatively weak band of PCR product was 427 base pairs (bp) long, representing a minority of gene copies was edited; and a band represents EDA positive FN gene at the length of 675 bp, as same as those isolated from untreated CNE-2Z or SW480 cells (Figure 1B). DNA sequencing confirmed that the 427 bp product lacks EDA exon, and the double strain breaks (DSBs) were repaired by non-homologous end-joining (NHEJ), by inserting 19 bp (Figure 1C).

### The exclusion of EDA exon inhibited cell proliferation and motility

**Figure 2:**
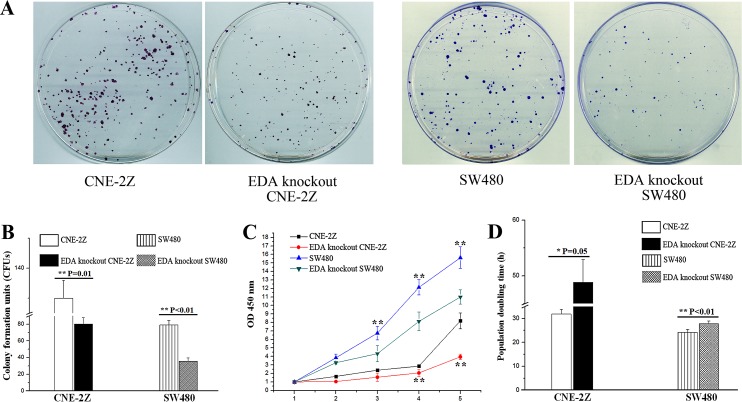
**(A)** CFUs developed from the untreated CNE-2Z or SW480 cells, in contrast with the EDA knockout CNE-2Z or SW480 cells respectively. **(B)** CFU numbers in untreated CNE-2Z or SW480, compared to the EDA knockout CNE-2Z or SW480 respectively. **(C)** Proliferation rates of untreated and EDA knockout CNE-2Z or SW480 cells. **(D)** PDT, measured using untreated and EDA knockout CNE-2Z (^*^P=0.05), as well as untreated and EDA knockout SW480 cells.

Contrary to the EDA over-expressing cells, which illustrated the pro-oncogenic effect (Supplementary Figures 1 to 3); EDA exclusion led to a decreased cell proliferation. As demonstrated by colony forming assay, EDA knockout were shown to generate a significantly lower number of colony formation units (CFUs) (80.30±7.50/400 cells in CNE-2Z & 35.67±4.04/400 cells in SW480), in comparison with that of the untreated CNE-2Z (130.00±6.00/400 cells, P=0.010) or SW480 cells (79.00±5.57/400 cells, P<0.001) (Figure 2A&2B). EDA knockout also led to prolonged population doubling time (PDT) (69.00±11.24 h in CNE-2Z & 27.80±0.58 h in SW480), in comparison with that of the untreated CNE-2Z (46.71±8.24 h, P=0.05) or SW480 cells (24.24±0.31 h, P=0.002) respectively (Figure 2C&2D), while the levels of Ki-67 decreased as well in each EDA knockout cell line (Figure 1A).

**Figure 3:**
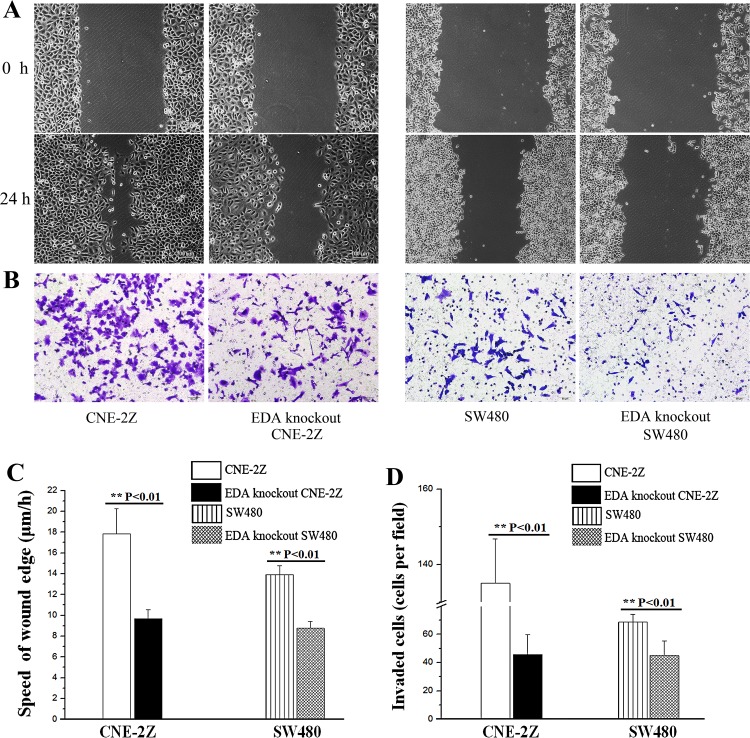
**(A)** Wound healing assay, showing the migratory abilities of untreated and EDA knockout cells. (original magnification, 100×; scale bar: 100 μm). **(B)** Transwell invasion assay results, showing the invasive rate of untreated and EDA knockout cells (original magnification, 200×; scale bar: 50 μm). **(C)** The speed of wound edge in the untreated and EDA knockout cells, including CNE-2Z and SW480. **(D)** The invasive rate of EDA knockout cells, compared with untreated CNE-2Z or SW480 cells, respectively.

In the wound healing assay, the speed of EDA knockout cells was shown to be 9.15±0.84 μm/h in CNE-2Z and 8.76±0.66 μm/h in SW480 cells, which was significantly lower than untreated controls (CNE-2Z: 17.84±2.4μm/h, P=0.005 & SW480: 13.91±0.86μm/h, P=0.001) (Figure 3A&3C). Similarly, the invasion rate of EDA knockout CNE-2Z (45.67±14.01 cells per field) and SW480 cells (45.00±10.44 cells per field) were significantly decreased, in comparison with that of untreated controls respectively (CNE-2Z: 135.00±11.79 cells per field, P=0.001 & SW480: 68.67±5.51 cells per field, P=0.003) (Figure 3B&3D).

### EDA knockout suppressed the epithelia-mesenchymal transition (EMT)

**Figure 4:**
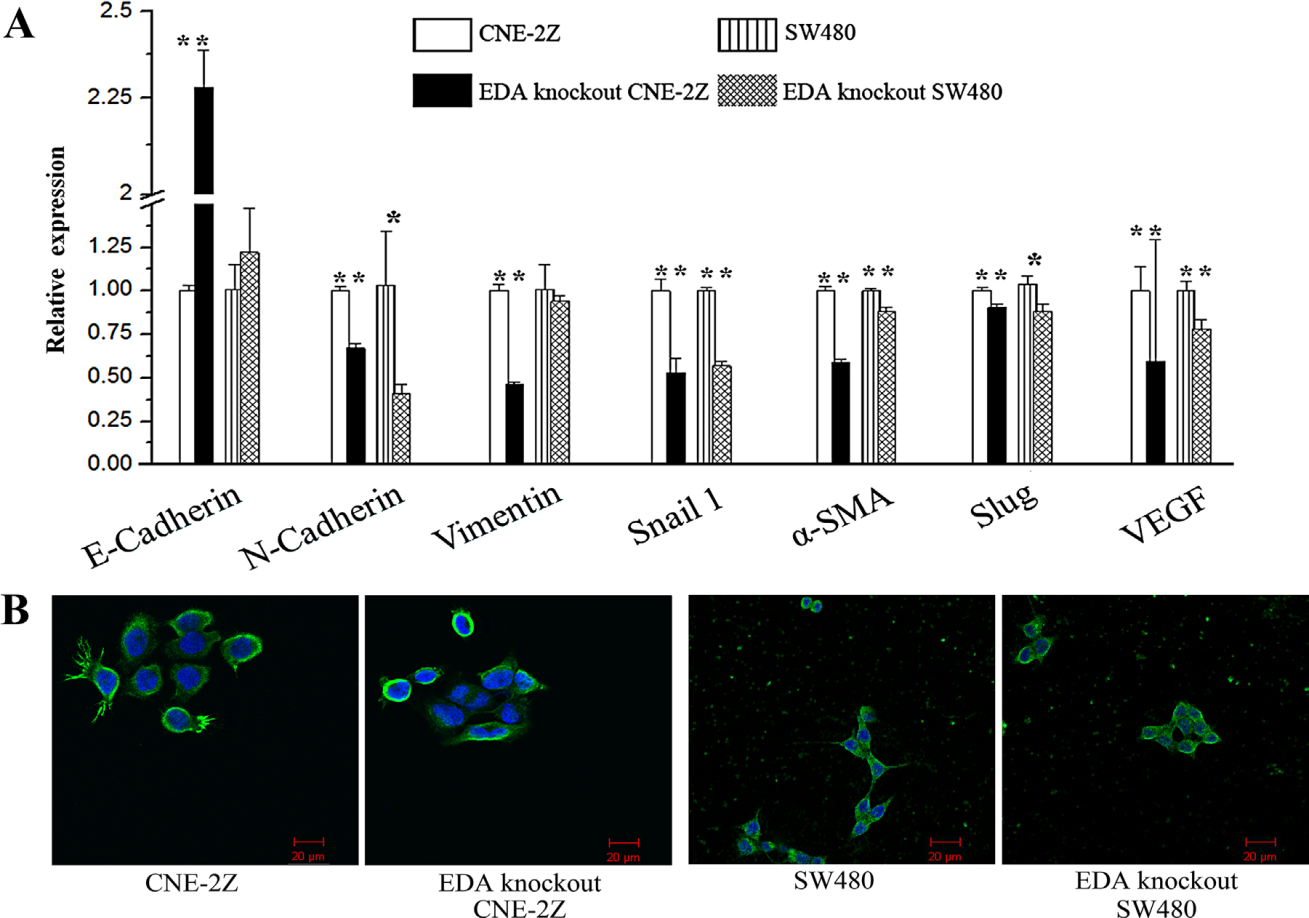
**(A)** Expression of epithelia-mesenchymal transtion (EMT)-related and *VEGF* genes in untreated and EDA knockout cells. **(B)** The Morphology and F-actin staining of CNE-2Z or SW480 cells, to illustrate the effects of EDA knockout on EMT (original magnification, 400×; scale bar: 20μm).

In CNE-2Z cells, EDA knockout led to the enhancement of E-cadherin and decrement of Snail 1 at both mRNA and protein levels, as well as the decreased N-cadherin, Vimentin, α-SMA, and Slug at mRNA level; consistently, the down-regulated mRNA and protein levels of Snail 1, and the decreased mRNA levels of N-cadherin, α-SMA and Slug, appeared in the EDA knockout SW480 cells as well. The mRNA levels of VEGF decline in both EDA knockout cell lines (Figure 1A & 4A) (Table 1).

**Table 1:** The normalized expression of genes in untreated and EDA knockout cells

Genes	untreated CNE-2Z	EDA knockout CNE-2Z	P
E-cadherin	1.0000±0.0318	2.2800±0.1070	^**^<0.001
N-cadherin	1.0000±0.0222	0.6700±0.0256	^**^<0.001
Vimentin	1.0000±0.0383	0.4645±0.0080	^**^<0.001
Snail1	1.0000±0.0651	0.5299±0.0828	^**^0.001
α-SMA	1.0000±0.0249	0.5900±0.0184	^**^<0.001
Slug	1.0000±0.0163	0.9044±0.0175	^**^0.002
VEGF	1.0000±0.0139	0.5935±0.0700	^**^0.001
	**untreated SW480**	**EDA knockout SW480**	**P**
E-cadherin	1.0065±0.1432	1.2209±0.2554	0.274
N-cadherin	1.0332±0.3119	0.4109±0.0501	^*^0.027
Vimentin	1.0066±0.1445	0.9387±0.0321	0.471
Snail1	1.0000±0.0206	0.5681±0.0233	^**^<0.001
α-SMA	1.0000±0.0147	0.8808±0.0264	^**^0.002
Slug	1.0369±0.0477	0.8835±0.0415	^*^0.014
VEGF	1.0010±0.0550	0.7804±0.0503	^**^0.007

^*^P < 0.05; ^**^P < 0.01.

Although the untreated CNE-2Z cells also displayed polygonal morphology, the EDA knockout CNE-2Z exhibited relatively weak and diffused F-actin staining, less membrane ruffling and lamellipodia. While the untreated SW480 tend to exhibit spindle morphology and more lamellipodia, in contrast with the polygonal morphology of EDA knockout SW480 (Figure 4B).

### *In vivo* EDA knockout using CRISPR/Cas9 system

**Figure 5:**
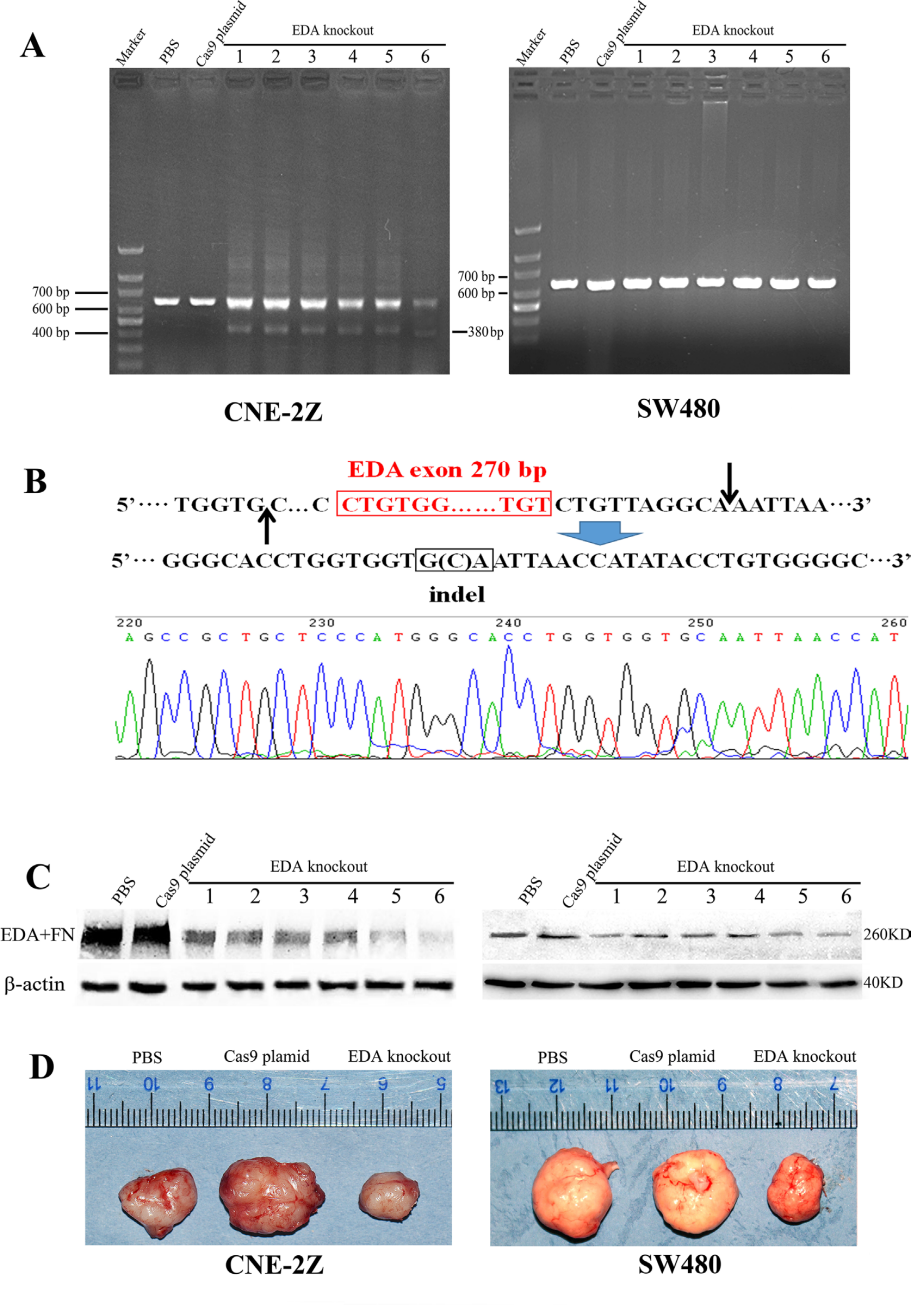
**(A)** The PCR products of the genome isolated from all EDA knockout samples, as well as the controls (PBS and Cas9 plasmid groups), CNE-2Z and SW480 xenografts were compared respectively. **(B)** The shorter PCR products (380 bp) representing another EDA knockout sequence in the 6th lane from the CNE-2Z xenografts were sequenced. **(C)** The protein level of EDA+FN in EDA knockout xenograft tumors and in the control groups, CNE-2Z and SW480 xenografts were compared respectively. **(D)** The size of the CNE-2Z or SW480 tumors between EDA knockout, PBS, and Cas9 groups.

After the establishment of the xenograft tumor models with CNE-2Z or SW480 cells, the CRISPR/Cas9 plasmids containing the sgRNA targeting EDA exon were injected into the tumors, while those samples injected with PBS or Cas9 plasmid without sgRNA were used as controls. PCR amplification was performed using total DNA isolated from tumor samples. In the xenograft tumors of CNE-2Z, EDA exon was shown to be removed at least partly *in vivo* (the 1st∼6th lane) (Figure 5A), and DNA sequencing confirmed that the DSBs were repaired by NHEJ. The gene sequences in most samples (the 1st∼5th lane) were identical to EDA negative FN gene identified *in vitro* (Figure 1B), however, in one sample (the 6th lane), DSBs were repaired at different sites, and only one base pair (bp) was inserted between the DNA breaks (Figure 5B), leading to a shorter PCR product in the 6th lane (380bp). A relatively small number of cells appeared affected by gene editing, according to the width and light intensity of PCR bands, however, the protein levels of EDA+FN considerably decreased in the EDA knockout CNE-2Z tumors (Figure 5C).

In the xenograft tumors of SW480, the PCR bands of EDA knockout gene seemed absent, suggesting a very low efficiency of gene editing *in vivo*, which perhaps overwhelmed by the overexposed EDA+FN bands (Figure 5A). But the obviously decreased protein of EDA+FN indicated the EDA exclusion still functioned (Figure 5C), suggesting the amplified effects from the gene editing to protein expression.

### EDA exclusion inhibits tumor progression *in vivo*

**Figure 6:**
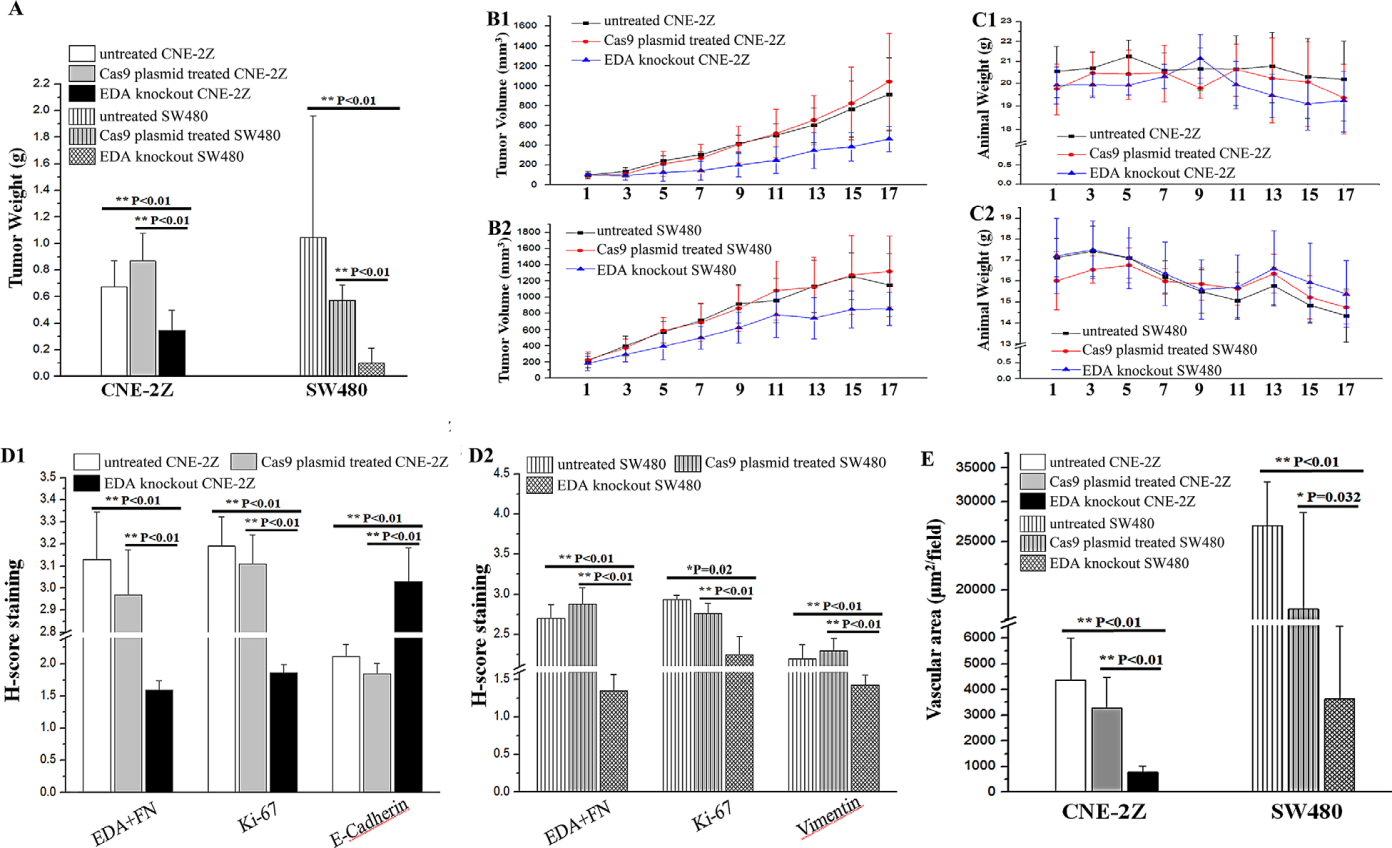
**(A)** The final weight in the EDA knockout CNE-2Z tumor (respectively compared with PBS or Cas9 plasmid groups), as well as the weight of EDA knockout SW480 tumor (compared with PBS or Cas9 plasmid groups respectively). **(B)** The growth rate of xenograft tumors in EDA knockout, PBS, and Cas9 plasmid groups, the CNE-2Z **(B1)** and SW480 **(B2)** tumor were presented respectively. **(C)** The changes of the animal weight in EDA knockout, PBS, and Cas9 plasmid groups, the animals carrying CNE-2Z **(C1)** and SW480 **(C2)** tumors were respectively exhibited. **(D)** The H-score of the immunohistochemical (IHC) staining of EDA+FN, Ki-67 and E-cadherin in CNE-2Z tumors **(D1)**; and that of EDA+FN, Ki-67, and Vimentin in SW480 tumors **(D2)**. **(E)** The vascular areas in EDA knockout group and PBS or Cas9 groups, accessed by CD34 staining of the endothelial cells, the samples of CNE-2Z and SW480 tumor were compared respectively.

At the final time point, the weight of CNE-2Z tumors were lower in the EDA knockout group (0.347±0.194 g), in comparison with that in the PBS (0.675±0.195 g; P=0.008) or Cas9 plasmid groups (0.867±0.208 g; P<0.001). Similarly, the weight of EDA knockout SW480 tumors were also less than their PBS (1.047±0.915 g; P<0.001) or Cas9 plasmid groups (0.572±0.117 g; P<0.001) (Figure 5D&6A). Actually, in comparison with control groups, the growth of CNE-2Z tumor was shown to be significantly inhibited from the 7th day following the EDA knockout (Figure 6B1 and Table 2); and the growth of SW480 tumor was inhibited from the 5th day until the termination (Figure 6B2 and Table 2). However, there was little difference in the animal weight between the EDA knockout and the control groups, either in the animals carrying CNE-2Z or SW480 xenograft tumors (Figure 6C1&6C2) (Table 3).

**Table 2:** The tumors volume along with the day after treatment

Days aftertreatment	Tumor Volume of CNE-2Z xenograft (mm^3^)			P		
	1(PBS)	2(Cas9 plasmid)	3(EDA knockout)	1/2	1/3	2/3
1	99.1350±22.1796	96.7567±31.8253	98.4850±39.0632	0.899	0.278	0.335
3	136.2017±37.8534	109.8100±34.7430	92.5933±44.7186	0.263	0.074	0.461
5	241.3767±47.47940	211.2333±127.1313	124.9750±88.0416	0.584	^*^0.047	0.131
7	303.9100±28.8846	268.6417±137.4934	142.1567±94.9973	0.542	^*^0.012	^*^0.041
9	415.3583±84.7677	405.5050±186.1797	198.2550±119.2779	0.902	^*^0.015	^*^0.019
11	499.1333±118.9764	517.3650±242.6938	248.1817±132.5145	0.858	^*^0.024	^*^0.017
13	601.5350±175.3146	651.5217±248.1406	345.6333±180.6527	0.677	^*^0.046	^*^0.02
15	763.7717±285.0559	821.3167±367.0476	383.2050±143.5666	0.728	^*^0.033	^*^0.016
17	912.0017±368.0845	1040.9917±486.9214	461.7217±126.7161	0.544	^*^0.047	^*^0.014
**Days aftertreatment**	**Tumor Volume of SW480 xenograft (mm^3^)**			**P**		
	**1(PBS)**	**2(Cas9 plasmid)**	**3(EDA knockout)**	**1/2**	**1/3**	**2/3**
1	215.7511±83.776	225.555±97.4038	181.3938±88.3222	0.826	0.442	0.319
3	397.9±120.8684	372.208±106.9834	292.265±92.2442	0.609	0.055	0.131
5	569.1644±129.8603	587.572±163.0796	392.07±164.959	0.786	^*^0.0200	^**^0.0090
7	711.7867±206.4703	688.213±234.3666	497.785±139.0018	0.801	^*^0.0380	0.057
9	919.1556±236.4647	861.386±282.7709	621.3775±194.6118	0.611	^*^0.0190	^*^0.0490
11	958.7944±272.8455	1081.733±361.7609	782.8625±280.5097	0.398	0.137	^*^0.0240
13	1134.6489±321.6374	1122.583±372.289	740.2638±256.7685	0.936	^*^0.0200	^*^0.0210
15	1258.9156±288.6477	1274.397±486.1922	848.2925±229.6154	0.927	^*^0.0290	^*^0.0210
17	1149.4178±391.0696	1318.356±438.2028	855.6438±207.8501	0.446	^**^0.0030	^*^0.0140

^*^P < 0.05; ^**^P < 0.01.

**Table 3:** The animals weight along with the day after treatment

Days after treatment	The Weight of animal carrying CNE-2Z xenograft (g)			P		
	1(PBS)	2(Cas9 plasmid)	3(EDA knockout)	1/2	1/3	2/3
1	20.5533±1.1915	19.7567±1.1362	19.925±0.8367	0.215	0.324	0.788
3	20.7083±0.7368	20.4633±1.0307	19.9383±0.5640	0.604	0.117	0.274
5	21.2700±0.7944	20.4300±1.1464	19.9133±0.43757	0.105	^*^0.014	0.306
7	20.5967±0.8355	20.4950±1.3178	20.3200±0.5557	0.856	0.624	0.756
9	20.6783±0.9959	19.7883±0.4465	21.1633±1.1629	0.376	^*^0.021	0.115
11	220.6550±1.6244	20.6400±1.2208	19.9467±1.0648	0.985	0.369	0.379
13	20.8017±1.6524	20.2350±1.9418	19.4683±0.9464	0.541	0.162	0.411
15	20.3083±1.8281	20.0583±1.9131	19.0950±1.1031	0.797	0.224	0.329
17	20.1883±1.8207	19.3600±1.5266	19.2330±1.3171	0.375	0.308	0.891
**Days after treatment**	**The Weight of animal carrying SW480 xenograft (g)**			**P**		
	**1(PBS)**	**2(Cas9 plasmid)**	**3(EDA knockout)**	**1/2**	**1/3**	**2/3**
1	17.1225±0.9145	16.013±1.3812	17.206±1.7903	0.117	0.904	0.076
3	17.428±1.1977	16.549±0.6462	17.486±1.3776	0.131	0.754	0.072
5	17.096±0.9621	16.756±0.8324	17.111±1.4783	0.505	0.976	0.487
7	16.193±0.7697	15.99±0.609	16.349±1.5125	0.666	0.74	0.447
9	15.5±1.0388	15.862±0.7697	15.596±1.4174	0.471	0.848	0.596
11	15.066±0.8319	15.641±0.7866	15.7133±1.5356	0.247	0.206	0.886
13	15.769±0.9022	16.35±0.926	16.5978±1.796	0.31	0.162	0.671
15	14.843±0.8335	15.224±1.0347	15.93±1.8866	0.52	0.082	0.251
17	14.35±1.2629	14.746±0.7994	15.3833±1.5933	0.484	0.083	0.276

^*^P < 0.05; ^**^P < 0.01.

**Figure 7:**
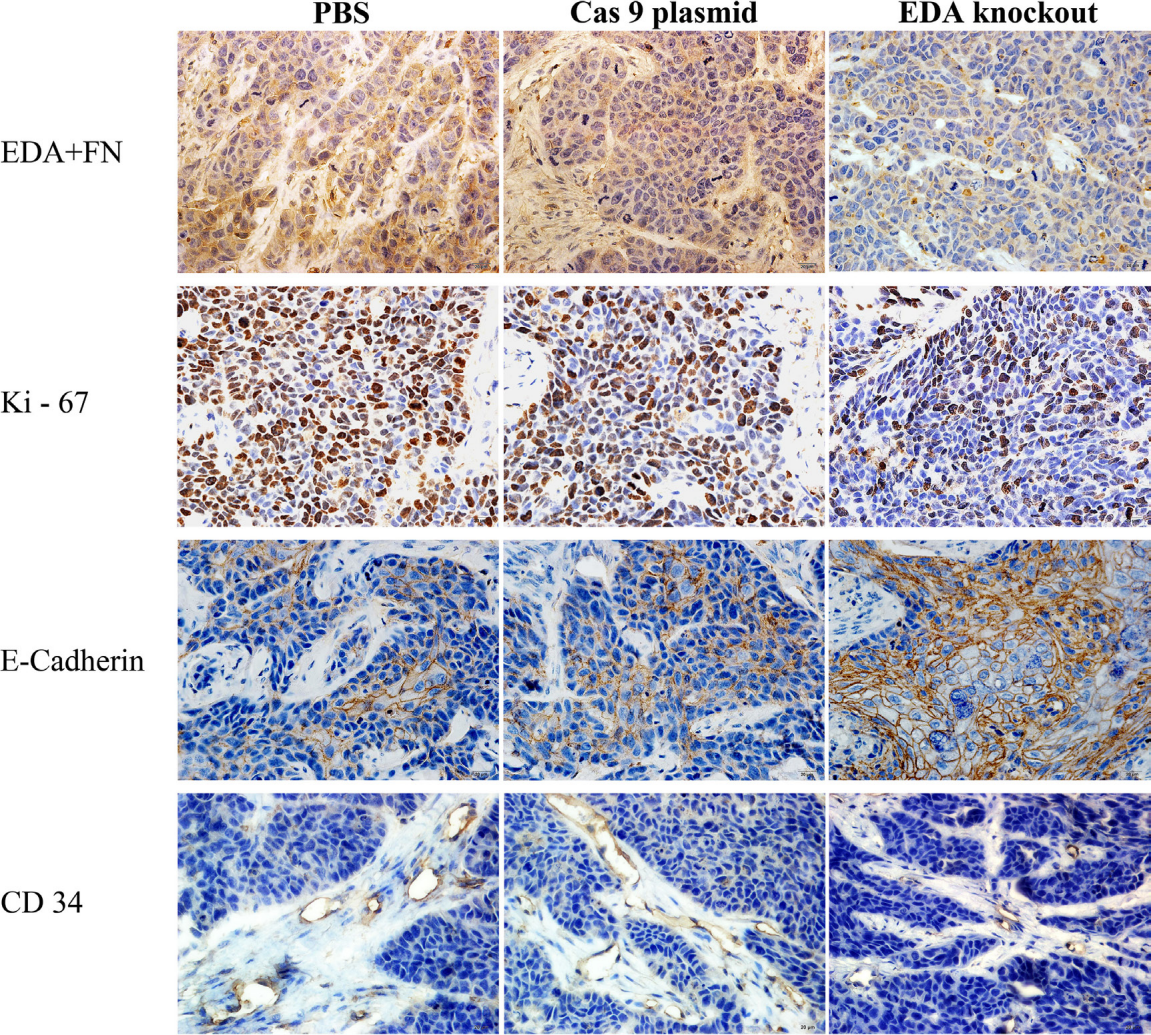
IHC EDA+FN, Ki-67, CD34, and E-cadherin staining of EDA knockout samples of CNE-2Z tumor, as well as the samples of PBS or Cas9 plasmid groups

The EDA+FN staining in the EDA knockout CNE-2Z tumor was significantly lower than that observed in the groups injected with PBS (P<0.001) or Cas9 plasmids without sgRNA (P<0.001). The Ki-67 staining was also lower than that in PBS (P<0.001) or Cas9 plasmid (P<0.001) groups; while the E-cadherin staining increased, in comparison with the PBS (P=0.001) or Cas9 plasmid groups (P<0.001) (Figure 6D1 & Figure 7). The vascular area in the EDA knockout xenografts was shown to be much smaller than that in PBS (P<0.001) or Cas9 plasmid groups (P<0.001), as demonstrated by CD34 staining of endothelial cells (Figure 6E) (Table 4).

**Table 4:** The Immunohistochemical staining of CNE-2Z and SW480 xenografts

Immunohistochemistry	CNE-2Z xenograft			P		
	1(PBS)	2(Cas9 plasmid)	3(EDA knockout)	1/2	1/3	2/3
EDA+FN (H-score)	3.13±0.214	2.97±0.204	1.59±0.194	0.345	^**^<0.001	^**^<0.001
Ki-67 (H-score)	3.11±0.131	3.19±0.132	1.86±0.127	0.4810	^**^<0.001	^**^<0.001
E-Cadherin (H-score)	2.11±0.195	1.84±0.164	3.13±0.153	0.0960	^**^0.001	^**^<0.001
Vascular area (μm^2^)	4378.15±1621.29	3275.72± 1201.42	781.26±250.02	0.2940	^**^<0.001	^**^<0.001
	**SW480 xenograft**			**P**		
	**1(PBS)**	**2(Cas9 plasmid)**	**3(EDA knockout)**	**1/2**	**1/3**	**2/3**
EDA+FN (H-score)	2.70±0.173	2.88±0.202	1.35±0.218	0.301	^**^<0.001	^**^<0.001
Ki-67 (H-score)	2.93±0.577	2.76±0.132	2.25±0.229	0.236	^*^0.02	^**^<0.001
E-Cadherin (H-score)	2.20±0.173	2.30±0.150	1.43±0.126	0.448	^**^0.001	^**^<0.001
Vascular area (μm^2^)	26895.35±5949.19	18356.45±10206.88	2831.91±3628.2	0.198	^**^<0.001	^*^0.032

^*^P < 0.05; ^**^P < 0.01.

**Figure 8:**
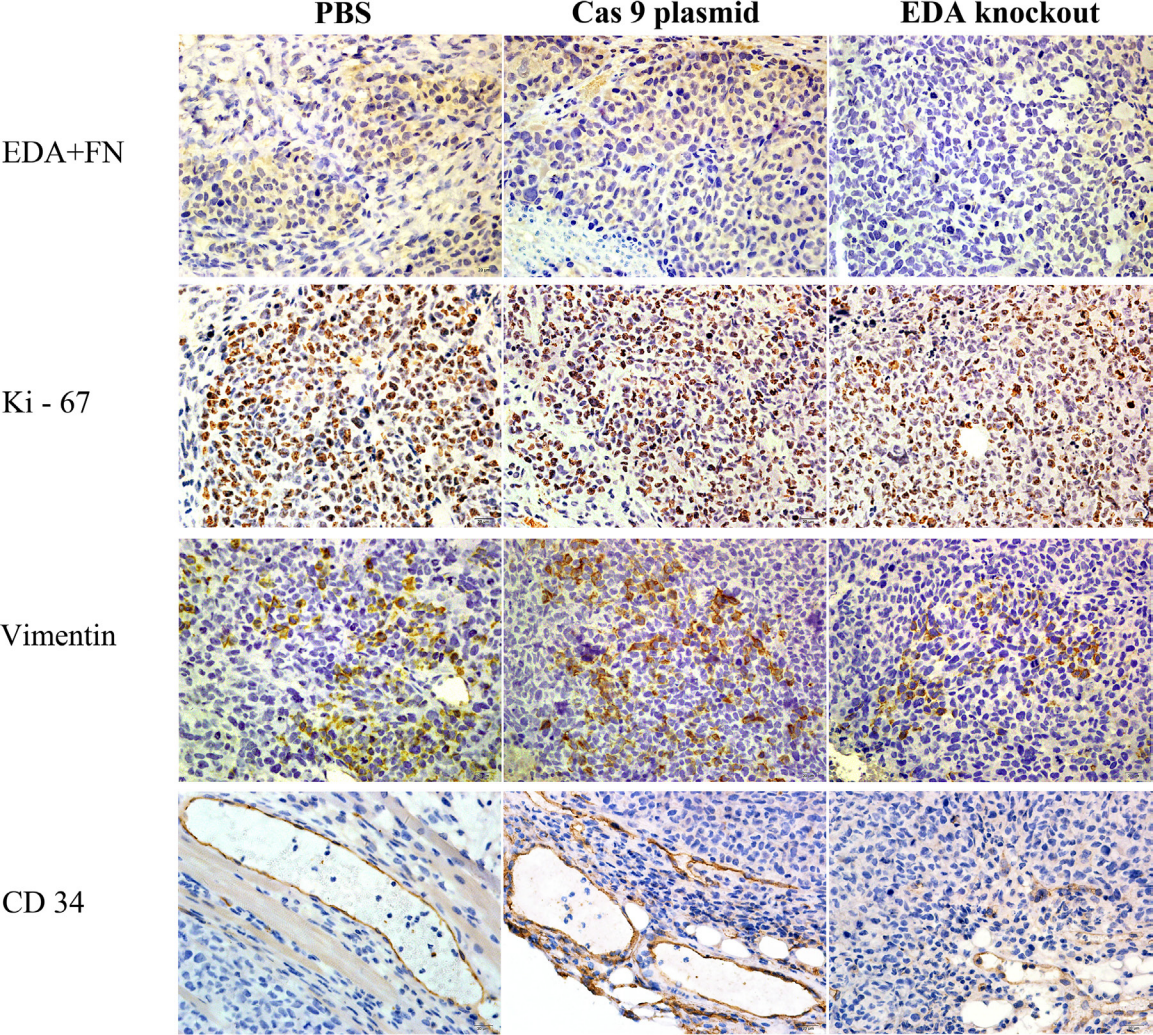
IHC EDA+FN, Ki-67, Vimentin and CD34 staining of EDA knockout sample of SW480 tumor, as well as the samples of PBS or Cas9 plasmid groups

In the tumors of SW480, EDA+FN staining was also significantly decreased in the knockout xenografts, in comparison with PBS (P<0.001) or Cas9 plasmids (P<0.001) groups. The Ki-67 staining in the knockout group was also lower than that in PBS (P=0.02) or Cas9 plasmid groups (P=0.008). The suppressed EMT seemed reflected in the decreased Vimentin staining in the knockout tumors, in contrast with that in PBS (P<0.001) or Cas9 plasmids (P<0.001) groups (Figure 6D2 & Figure 8). The EDA knockout xenografts of SW480 developed less vascular area than that in PBS (P<0.001) or Cas9 plasmid groups (P=0.032) (Figure 6E) (Table 4).

## DISCUSSION

In this study, nasopharyngeal cell line CNE-2Z and colorectal carcinoma cell line SW480 were selected for their survival relying on EDA and easiness to form xenograft tumors [18, 19]. The promotion of cell motility of EDA had been demonstrated in salivary adenoid cystic carcinoma (SACC) previously [15], it suggested the necessity of ECM in the development of cancers [11, 20]. On this basis, we eliminated EDA exon in CNE-2Z and SW480 cell lines *in vitro*. According to the width and light intensity of PCR products, only minority of gene copies were edited, but EDA+FN protein levels considerably decreased in both cell lines. This may be explained by the ability of sgRNA-Cas9 complex to access the uncoiled DNA during gene replication or transcription, which is present in dividing cells or the actively transcribed genes [21, 22]. Therefore, in the subpopulation of cells that are actively proliferating or transcribing FN genes, the EDA exon tend to be knockout efficiently [13], allowing an amplified effects on the ECM components, thus leading to the decreased tumor cell propagation. In previous study, the highly aggressiveness but slow growth of SACC [23], made the cell motility relied on EDA significantly, but little effects of EDA on the cell proliferation [15]; however, in this study, EDA knockout inhibited both the motility and proliferation of CNE-2Z or SW480, consistent to the growth characteristics [18, 19]. Taken together, EDA knockout with CRISPR/Cas system provide a strategy for interrupting the interaction of tumor cells with their microenvironment, instead of eradicating cancer cells themselves.

The strategies for cancer treatment involving the complete eradication of tumor cells remain the mainstream in the field of cancer research. However, these approaches are limited by a series of problems, including the development of drug resistance or the immune evasion of tumor cells, as well as cancer recurrence and metastases [24–26]. Any attempts to eradicate all tumor cells may impose a survival pressure on tumor cells, which may force the tumor cells to disseminate and lead to the selection of drug insensitive subpopulation [1, 2, 26]. For these reasons, the remaining cancer cells can survive the initially effective treatment, and tumor may resume progression [5, 6]. A strategy based on the inhibition of tumor progression instead of eradicating cancer cells may be a feasible way to prolong patient survival, in a way similar to that of SACC patients, where the early development of metastases prevents successful tumor removal, but patient survival time is measured in years, due to the slow tumor growth [23]. Additionally, although an initial high dose of paclitaxel leads to the reduction in tumor volume in animal models, this is followed by a rapid cancer progression; however, the application of lower doses of paclitaxel led to a continuous inhibition of tumor growth, consequently prolonging survival periods [14]. The inhibition rather than eradication, may relieve the survival pressure and artificial selection, it may facilitate to maintain the intra-tumor heterogeneity, and hence the intercellular competition is likely to further inhibit tumor progression [13, 27].

**Figure 9:**
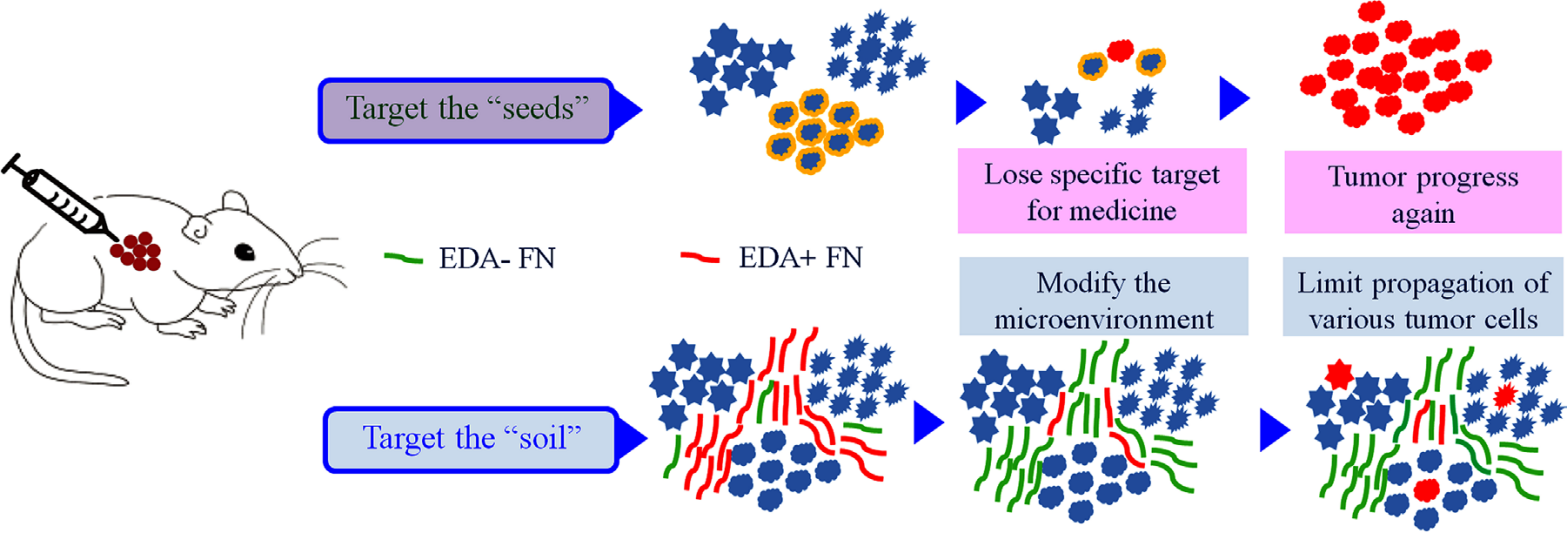
A schematic representation of treatment strategies Using the strategy focusing on the eradication of tumor cells (the “seeds”), the therapeutic evasion appears inevitable. Alternatively, by targeting tumor microenvironment (the “soil”), tumor progression may be prevented without leading to the drug resistance development, as well as maintaining the heterogeneity within tumors.

Since the evolution of a ecosystem is affected by the environmental conditions, the modified ECM components may be an unfavorable factor on the tumor growth and progression (Figure 9) [8]. To confirm this hypothesis, CRISPR/Cas9 system was applied *in vivo*, knocking out EDA exon of the FN gene in the xenograft tumors. In accordance with the previous *in vitro* experiments, the relatively low efficiency of gene editing *in vivo* was accompanied by considerable decrease in EDA+FN protein levels, and significantly inhibited tumor growth of CNE-2Z or SW480 xenografts. While the observed changes in animal weight were similar in all investigated groups, indicating a low toxicity of the applied method. The declined cell proliferation most likely led to the initial inhibition of tumor growth, as demonstrated by a decrease in Ki-67 staining intensity in the EDA knockout group. Additionally, an increase in E-cadherin expression in the CNE-2Z tumor may limit the propagation of EDA knockout tumors; while the decreased Vimentin in the SW480 tumor suggested impaired cell propagation. Since EDA+FN has various paracrine and autocrine effects, the EDA-FN is likely to act as the physical limits of tumor cell migration rather than the pro-oncogenic roles [12], the gene editing affects not only the modified cells themselves, but also the entire tumor [8, 9]. The exclusion of EDA decreased VEGF expression, as investigated *in vitro*, which decreases angiogenesis or vascularization *in vivo*, may amplified the inhibition of tumor progression [28, 29].

The low efficiency of *in vivo* genome editing, even when using viral vector [21, 30], limits the application of CRISPR/Cas system [17], since the greater number of inaccessible cells may offset the effects of modifying onco-genes in some easily accessible cells [16, 31–33]. This issue may be avoided by targeting molecules that constitute tumor microenvironment, which would allow CRISPR/Cas system to access the paracrine or autocrine effects of subpopulation responsible for the whole tumor progression, just as the altered EDA+FN and VEGF in this study.

In various tumors, EDA exon knockout led to significantly inhibited tumor progression with little side effects *in vivo*. It suggested that, by modifying ECM components constituting tumor microenvironment, we may avoid the issue of the low efficiency of *in vivo* gene editing; and the general reliance of various tumor cells on some components of ECM made the specific therapeutic markers unnecessary [5, 6]; furthermore, the inhibition instead of eradication imposes little survival pressure on the tumor cells and facilitate a continuously inhibitive effects [14].

## MATERIALS AND METHODS

### Cell culture

Poorly differentiated human nasopharyngeal carcinoma cell line CNE-2Z was purchased from China Infrastructure of Cell Line Resource (Beijing, China), and it was grown in RPMI 1640 medium (Gibco, Grand Island, NY, USA). The colorectal carcinoma cell line SW480 was preserved in Peking University School of Stomatology, it was grown in Dulbecco's Modified Eagle's Medium (DMEM) (Gibco, Grand Island, NY, USA). All the cells were cultured in medium containing 10% fetal bovine serum (FBS, Gibco) at 37°C and 5% CO_2_.

### Transfection with Cas9 plasmids containing single guide (sg) RNAs

Two single guide (sg) RNAs, complementing the sequences flanking EDA exon, were designed and cloned into CRIPSR/Cas9 plasmid PX330, as described previously [15, 17, 34].

As the CNE-2Z cells reached about 60-70% confluence, and the SW480 reach about 70-80% confluence, the medium were replaced with serum free one for 6 h. Then the two plasmids were co-transfected into the cells respectively, with the Lipofectamine 2000 (Life Technologies). In 24 hours later, puromycin was added to CNE-2Z cells; while in the SW480 cells, puromycin was added at 36 hours after transfection. Both cell lines were incubated with puromycin (1.6 μg/ml) for additional 48 h.

The genomic DNA was extracted, and then the efficiency of EDA knockout was accessed with PCR, DNA sequencing was used to confirm EDA knockout [15, 35]. Untreated CNE-2Z or SW480 cells were used as control respectively. The sequences of primer and sgRNAs are presented in Table 5.

**Table 5:** Sequences of CRISPR sgRNA and confirming primers used in this study

Name	sgRNA sequence (5′-3′)	PAM sequences(5′-3′)	DSB site in fibronectin (FN)genome (ref|NC_018913.2|)
sgRNA up-stream-F	GTTACAGACATTGATCGCCCTAA	CAG	216251681
sgRNA up-stream-R	AACTTAGGGCGATCAATGTCTGT		
sgRNA down-stream-F	GTTCTGATTGGAACCCAGTCCAC	AGG	216251434
sgRNA down-stream-R	AACGTGGA CTGGGTTCCAATCAG		
	primers	productcontaining EDA	product without EDA
Primer-down	atagtgggttaattggact	675bp	≈400bp
Primer-up	agggtaatcacagggag		

### Colony-forming unit (CFU) and population doubling time (PDT) assays

EDA-knockout and untreated CNE-2Z or SW480 cells were seeded onto 100-mm dishes at the density of 400 cells/dish for the CFU assay. The cells were cultured in RPMI 1640 or DMEM for 7 days and stained with 0.1% crystal violet, respectively. Aggregations of more than 50 cells were defined as colony formation units (CFUs).

Additionally, the two cell lines seeded onto 96-well plates at a density of 1000 cells/well respectively, for the determination of PDT. The cell numbers were determined daily in six wells, using Cell Counting Kit-8 (Dojindo, Kumamoto, Japan), according to the manufacturer's instructions.

### Wound-healing and transwell invasion assays

Cells were seeded into 24-well plates, grown to confluence, synchronized in fresh medium containing 0.5% FBS for 6 h, and the cell monolayer was wounded using a 300-400 μm pipette tip. The average linear speed of the movement of wound edges was quantified over 24 h. Cell invasion assay was performed using transwell chambers with a polycarbonate membrane (Millipore, Bedford, MA, USA), coated with 20 μg ECM gel (Sigma-Aldrich, Trading Co Ltd, Shanghai, China). In the upper chambers, CNE-2Z was seeded at 4×10^4^ cells/well, and SW480 was seed at 1×10^5^ cells/well. After 24 h of incubation, the membranes were stained with 0.1% crystal violet and the cells remaining on the upper surface of the membrane were wiped off.

### Establishment of the xenograft model and *in vivo* EDA knockout using CRIPSR/Cas9 system

Four-week-old male BALB/c nude mice were purchased from Vital River Experimental Animal Technique Company (Beijing, China) and maintained in a specific pathogen-free condition. All the animals were acclimatized for 1 week before experiments, and then maintained under controlled temperature (22±2°C), with light dark periods of 12 hours and with free access to water and commercial diet [36].

The cells were digested using trypsin and washed by phosphate buffered saline (PBS) twice respectively, the CNE-2Z was re-suspended at the concentration of 3×10^7^cells/ml in PBS; the concentration of SW480 was 5×10^7^cells/ml. Following this, 3×10^6^ CNE-2Z cells as well as 5×10^6^ SW480 cells were subcutaneously injected into the flank region of each mouse, respectively. Tumor volume was measured with caliper and calculated using the following formula: V=length×width^2^×(π/6) [37]. This study was approved by the animal care committee guidelines of the Peking University biomedical ethics committee for laboratory animal welfare ethics, Bejing, China (Permit number: LA2012-53).

When the subcutaneous tumor volumes reached 70-100mm^3^, mice were randomly divided into three groups (n=6). CRISPR/Cas9 plasmids with sgRNAs for EDA knockout, were delivered by intra-tumor injections every 2 days (1μg/μL, 40μL/mouse; EDA knockout group), and the mice with tumors injected with PBS (40μL/mouse) or the CRISPR/Cas9 plasmid without sgRNAs (1μg/μL, 40μL/mouse) were included in the control groups: PBS and Cas9 plasmid groups, respectively. Tumor size and mouse weights were measured every other day. The mice were euthanized at the 17th day, and the weights of tumors were recorded.

### RNA extraction, reverse transcription, and PCR amplification

Total RNA was isolated from all cells with TRIZOL reagent (Life Technologies), and 2-μg samples were reverse-transcribed into cDNA using the SuperScript First-Strand Synthesis System (Life Technologies), according to manufacturer's instructions. These reactions were performed in a 20-μL reaction mixture with ABI 7500 real-time PCR system (ABI), including an initial incubation at 95°C for 10 min, followed by 40 cycles of annealing/extension at 60°C for 1 min, and denaturation at 95°C for 15 s. The expression of E-cadherin, N-cadherin, Vimentin, Snail1, alpha-smooth muscle actin (α-SMA), Slug and VEGF was determined and normalized with human β-actin. All primers used in these experiments are described in Table 6.

**Table 6:** The primers used for real-time PCR

Name	Primer	Sequence(5′-3′)	Gene ID
E-cadherin	Forward	AACGAGGCTAACGTCGTAATCA	NM_004360.3
	Reverse	CCCAGGGGACAAGGGTATGAA	
N-cadherin	Forward	GAGATCCTACTGGACGGTTCG	NM_001792.3
	Reverse	TCTTGGCGAATGATCTTAGGA	
Vimentin	Forward	AAGGCGAGGAGAGCAGGATT	NM_003380.3
	Reverse	GGTCATCGTGATGCTGAGAAG	
Snail1	Forward	GCCTTCAACTGCAAATACTGC	NM_005985.3
	Reverse	CTTCTTGACATCTGAGTGGGTC	
α-SMA	Forward	AAAAGACAGCTACGTGGGTGA	NM_001141945.1
	Reverse	GCCATGTTCTATCGGGTACTTC	
Slug	Forward	GAGCATTTGCAGACAGGTCA	NM_005985.3
	Reverse	CCTCATGTTTGTGCAGGAGA	
VEGF	Forward	TTATGCGGATCAAACCTCACC	NM_001171630.1
	Reverse	GAAGCTCATCTCTCCTATGTGC	
β-actin	Forward	CATGTACGGTTGCTATCCAGGC	NM_001101.3
	Reverse	CTCCTTAATGTCACGCACGAT	

### Western blot analysis

Total proteins were extracted from cells grown in complete medium and the supernatants collected from 24-h serum-deprived cells [15]. Proteins were separated on a 12% SDS-PAGE, and transferred to nitrocellulose filter membranes, which were probed with the following antibodies overnight at 4°C: anti-E-cadherin (Santa Cruz Biotechnology, Dallas, TX, USA), anti-Snail1 (Cell Signaling Technology, Danvers, MA, USA), as well as anti-Flag, anti-Vimentin, IST-9, anti-FN, anti-Ki-67, and anti-β-actin (Abcam Ltd., Cambridge, MA, USA) antibodies. Immunocomplexes were detected with an enhanced chemiluminescence blotting kit (Applygen Technology Inc., Beijing, China).

### Immunofluorescence and immunohistochemical (IHC) staining

Cells were fixed in 95% ethanol, blocked in 1% BSA in PBS, permeabilized in 0.5% Triton X-100 in PBS, and stained with anti-F-actin monoclonal antibody (1/500; Abcam Ltd., Cambridge, MA, USA) at 4°C overnight. F-actin was detected by using the indirect immunofluorescence, and fluorescence images were obtained using the DAPI excitation settings on the laser confocal microscope (Lsm 5 Exciter; Zeiss, Jena, Germany).

Xenograft tumor tissues were sectioned into 4-μm-thick slices and stained with IST-9, anti-EDA+FN, anti-Ki-67, anti-CD34, and anti-E-cadherin (Abcam Ltd., Cambridge, MA, USA) antibodies at 4°C overnight. They were subsequently incubated with the biotinylated secondary antibody (1:200) for 1 h. The immunocomplexes were visualized with diaminobenzidine (Zhongshan Golden Bridge Biological Technology CO., LTD, Beijing, China). Following the IHC staining for EDA+FN, Ki-67, and E-cadherin, the obtained results were semi-quantitatively analyzed by histological score (H-score) based on the positively stained cell number and staining intensity. The H-score was calculated using the following formula: HS=Σ Pi (1+i)/100, where Pi represents the percentage of stained cells at each intensity score (0-3) [15, 38]. The area of microvessels (μm^2^), identified by CD34 staining of vascular endothelial cells was assessed as previously described [29].

### Statistical analysis

Data obtained *in vitro* was compared between the EDA knockout and untreated cells. All experiments were performed in triplicates. Quantitative data were expressed as mean ± standard deviation (SD) and analyzed using the Student's *t*-test, for the differences between paired groups.

*In vivo* obtained results were compared between PBS, Cas9 plasmid, and EDA knockout groups. Each group contained six biological samples. H-scores obtained by IHC analyses, the area of microvessels, tumor volumes and the weights of tumor or animal were expressed as mean ± SD, and they were analyzed using one-way analysis of variance (ANOVA) for the difference between these groups. Statistical significance was set at P<0.05.

## SUPPLEMENTARY FIGURES



## Abbreviations

EDA+FN: extra domain A positive fibronectin
CRISPR/Cas system: clustered regularly interspaced palindromic repeats-associated system
ECM: extracellular matrix
VEGF: vascular endothelial growth factor
EMT: epithelial-mesenchymal transition
SACC: salivary adenoid cystic carcinoma
α-SMA: alpha-smooth muscle actin
