# Supplementary material for: Gene editing of the extra domain A positive fibronectin in various tumors, amplified the effects of CRISPR/Cas system on the inhibition of tumor progression
Source: Oncotarget. 2017 Sep 21;8(62):105020–36. doi: 10.18632/oncotarget.21136 (PMC5739617; doi:10.18632/oncotarget.21136)
Supplement: Supplementary file 1 [file oncotarget-08-105020-s001.pdf]

# Gene editing of the extra domain A positive fibronectin in various tumors, amplified the effects of CRISPR/Cas system on the inhibition of tumor progression

## SUPPLEMENTARY MATERIALS

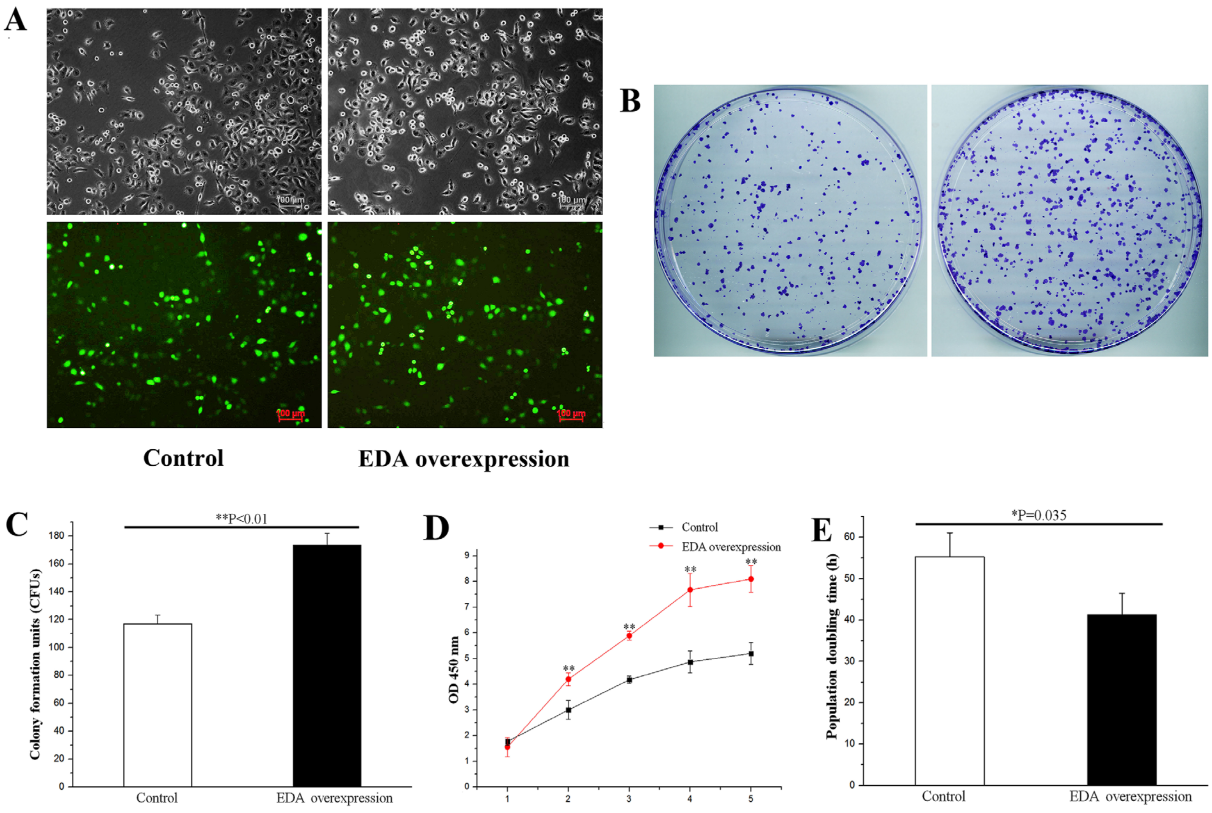


**Supplementary Figure 1: (A)** Transfection efficiency of the plasmid encoding recombinant extra domain A (EDA) (right), and the control plasmid (left), in the CNE-2Z cells (original magnification, 100×; scale bar: 100 μm). **(B)** The colony formation units (CFUs) developed from the EDA overexpressing (right) and the control CNE-2Z cells (left). CFUs were stained with 0.1% crystal violet. **(C)** CFUs developed from EDA overexpressing CNE-2Z cells (**P=0.001, compared with the control). **(D)** Proliferation rates of EDA overexpressing and control CNE-2Z cells. **(E)** The population doubling time (PDT) of the EDA overexpression CNE-2Z cells (*P=0.035, in comparison with the control).

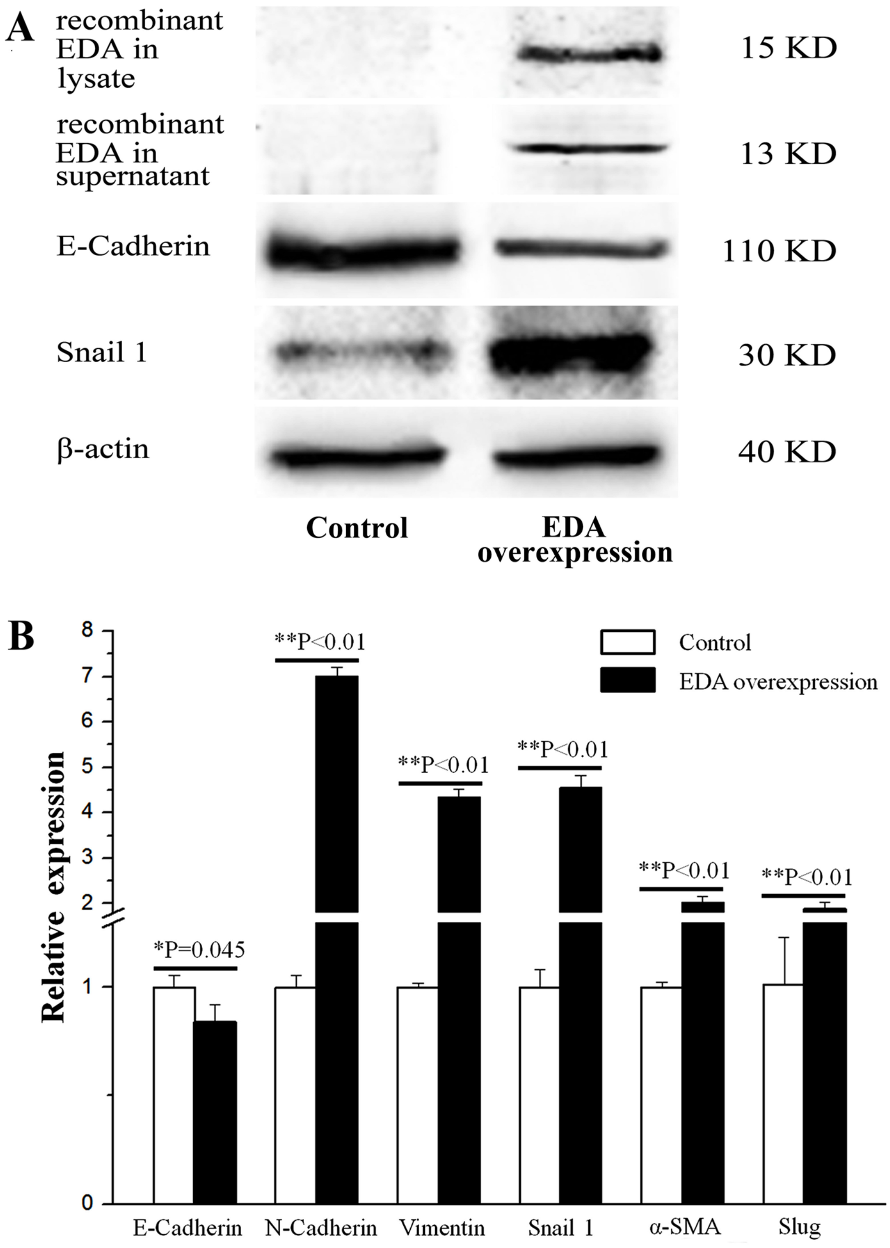


**Supplementary Figure 2: (A)** Western blot analysis of recombinant EDA, E-cadherin, and Snail 1 expression in the CNE-2Z cells. **(B)** Epithelia-mesenchymal transition (EMT) related genes expression alterations in EDA overexpressing and the control CNE-2Z cells.

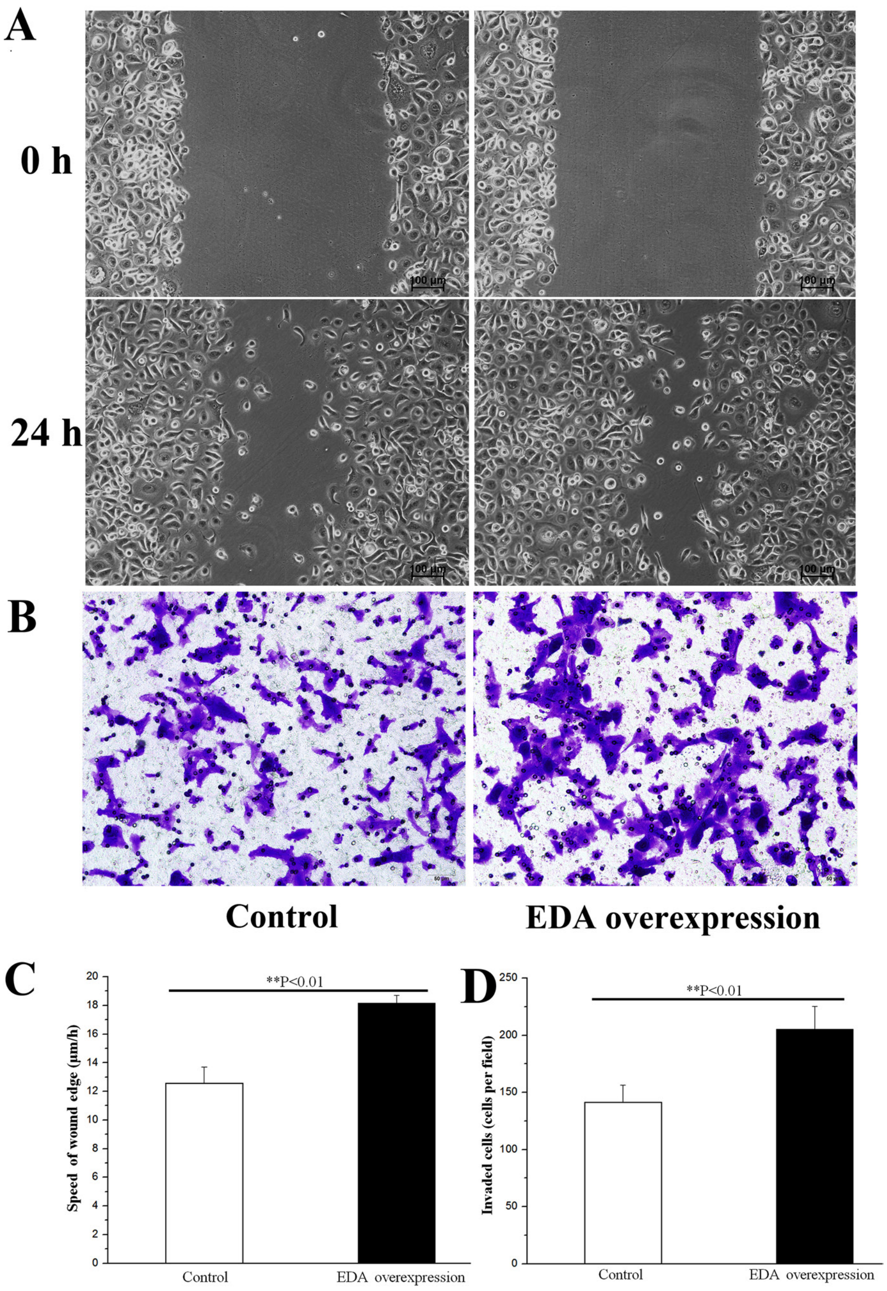


**Supplementary Figure 3: (A)** Wound healing assay, using EDA overexpressing (right) or control CNE-2Z cells (left), during 24 h (original magnification, 100×; scale bar: 100 μm). **(B)** The number of EDA overexpressing (right) or control (left) CNE-2Z cells that invaded through the transwell insert membranes (original magnification, 200×; scale bar: 50 μm). **(C)** Wound healing assay, showing the increase in wound healing ability of the EDA overexpressing CNE-2Z cells ($^{**}P=0.001$, compared with the control). **(D)** Invasion rate of EDA overexpressing CNE-2Z cells ($^{**}P<0.001$, compared with the control).
